# Supplementary material for: MetaRibo-Seq measures translation in microbiomes
Source: Nat Commun. 2020 Jun 29;11:3268. doi: 10.1038/s41467-020-17081-z (PMC7324362; doi:10.1038/s41467-020-17081-z)
Supplement: Supplementary file 10 — Supplementary Data 7 [file 41467_2020_17081_MOESM10_ESM.zip › File2/Confidence_High_Taxonomy/260003_out.krona.html]

Javascript must be enabled to view this page.

members
magnitude
magnitudeUnassigned
count
unassigned
taxon
rank

260003\_out

17

superkingdom
2
17

phylum
1239
17

186801
class
17

17
186802
order

species
1898207

SRS011134\_contig\_number\_3038SRS011302\_contig\_number\_26870SRS013476\_contig\_number\_11518SRS020394\_contig\_number\_18310SRS020869\_contig\_number\_contig-100\_25820.110540SRS024132\_contig\_number\_45175SRS048870\_contig\_number\_5067SRS052697\_contig\_number\_10108SRS064757\_contig\_number\_18826SRS1041033\_contig\_number\_29204SRS1041137\_contig\_number\_contig-100\_192.169887SRS104636\_contig\_number\_2879SRS144135\_contig\_number\_13659SRS146812\_contig\_number\_59485SRS147139\_contig\_number\_24638SRS147557\_contig\_number\_23856SRS150029\_contig\_number\_12732
17
